# Immunogenicity of Two Versus Three Doses of Hepatitis B Vaccine When Administered to Children Aged 2–18 Months: A Randomized Clinical Trial

**DOI:** 10.1093/infdis/jiag122

**Published:** 2026-02-25

**Authors:** Koffi Barnabé Kegbevi, Yossi Febriani, Chantal Sauvageau, Marie-Michelle Ursu, Caroline Quach, Michaël Desjardins, Julie Bestman-Smith, Vladimir Gilca, Nicholas Brousseau

**Affiliations:** Département de médecine sociale et préventive, Faculté de médecine, Université Laval, Québec, Québec, Canada; Axe maladies infectieuses et immunitaires, Centre de Recherche du Centre Hospitalier Universitaire de Québec-Université Laval, Québec, Québec, Canada; Axe maladies infectieuses et immunitaires, Centre de Recherche du Centre Hospitalier Universitaire de Québec-Université Laval, Québec, Québec, Canada; Département de médecine sociale et préventive, Faculté de médecine, Université Laval, Québec, Québec, Canada; Axe maladies infectieuses et immunitaires, Centre de Recherche du Centre Hospitalier Universitaire de Québec-Université Laval, Québec, Québec, Canada; Direction des risques biologiques, Institut national de santé publique du Québec, Québec, Québec, Canada; Département de microbiologie, infectiologie et immunologie, Faculté de médecine, Université de Montréal, Montréal, Québec, Canada; Axe maladies infectieuses et soins aigus, Centre de recherche Azrieli du CHU Sainte-Justine, Montréal, Québec, Canada; Département de microbiologie, infectiologie et immunologie, Faculté de médecine, Université de Montréal, Montréal, Québec, Canada; Axe maladies infectieuses et soins aigus, Centre de recherche Azrieli du CHU Sainte-Justine, Montréal, Québec, Canada; Département de microbiologie, infectiologie et immunologie, Faculté de médecine, Université de Montréal, Montréal, Québec, Canada; Axe immunopathologie, Centre de recherche du Centre hospitalier de l’Université de Montréal, Montréal, Québec, Canada; Axe maladies infectieuses et immunitaires, Centre de Recherche du Centre Hospitalier Universitaire de Québec-Université Laval, Québec, Québec, Canada; Direction des risques biologiques, Institut national de santé publique du Québec, Québec, Québec, Canada; Département de médecine sociale et préventive, Faculté de médecine, Université Laval, Québec, Québec, Canada; Axe maladies infectieuses et immunitaires, Centre de Recherche du Centre Hospitalier Universitaire de Québec-Université Laval, Québec, Québec, Canada; Direction des risques biologiques, Institut national de santé publique du Québec, Québec, Québec, Canada

**Keywords:** clinical trial, hepatitis B vaccines, reduced-dose schedule, immunization, infants

## Abstract

**Background:**

A 2-dose hepatitis B vaccination schedule is highly immunogenic in children aged ≥1 year, but data for infants are scarce. Our goal was to compare the immunogenicity of 2- and 3-dose hepatitis B vaccination schedules in this population.

**Methods:**

Children in the experimental group were recruited at 2 months of age and randomized to receive a homologous (Infanrix-hexa/Infanrix-hexa) or heterologous (Infanrix-hexa/Twinrix) 2-dose schedule at 2 and 12 months. Children in the control group were recruited at 18 months and had received a homologous 3-dose schedule (Infanrix-hexa/Infanrix-hexa/Infanrix-hexa) at 2, 4, and 18 months. All groups received a challenge dose (Twinrix) 3 years later.

**Results:**

One month after the primary series, seroprotection rates (anti-HBs ≥10 mIU/mL) were high and similar among the 3 groups (heterologous 2-dose, 91.7%; homologous 2-dose, 90.9%; 3-dose, 94.2%). Geometric mean titers (GMTs) were lower in the 2-dose groups than in the 3-dose group. Post-challenge dose, the majority exhibited an anamnestic response, similar across all 3 groups (heterologous 2-dose, 97.2%; homologous 2-dose, 95.5%; 3-dose, 92.7%). GMTs were numerically higher in the heterologous 2-dose group (9380.5 mIU/mL) than in the 3-dose group (6900.6 mIU/mL) (*P* = .4). The proportion exhibiting local reactions was significantly lower after the heterologous 2-dose schedule than after the homologous 2-dose schedule.

**Conclusions:**

The anamnestic response 3 years after 2-dose (2 and 12 months) and 3-dose (2, 4, and 18 months) hepatitis B vaccination schedules was similar. The heterologous 2-dose schedule was more immunogenic and less reactogenic than the homologous 2-dose schedule.

## Teaser

The response to a challenge dose 3 years after 2-dose (2 and 12 months) and 3-dose (2, 4, and 18 months) hepatitis B vaccination schedules was similar. A heterologous 2-dose schedule was more immunogenic and less reactogenic than a homologous 2-dose schedule.

Hepatitis B (HB) remains a major public health concern. The World Health Organization (WHO) estimated that 900 000 deaths were caused by hepatitis B virus (HBV) in 2022 [1], despite it being preventable through vaccination. A 3-dose schedule is recommended by the WHO for universal infant vaccination [2], offering very high protection [3].

Studies have compared a 2-dose with a 3-dose hepatitis B vaccination schedule and concluded that both schedules had similar immunogenicity in adolescents, pre-adolescents, and children over 1 year of age [4–6]. A 2-dose schedule has been approved in some countries for children aged 1 year or older [7, 8] or for adults (new HEPLISAV-B vaccine) [9]. However, studies evaluating the immunogenicity of a 2-dose schedule in children under 1 year of age are very limited and include few children [10–12].

After using a 3-dose schedule for hepatitis B in grade 4 students, the province of Quebec was the first Canadian province to evaluate and implement a 2-dose schedule for these same children [13–16]. In 2013, following the approval of new combination vaccines, Quebec changed its recommendations and implemented a 3-dose schedule at 2, 4, and 18 months with the hexavalent vaccine Infanrix-hexa (DTaP-IPV-Hib-HB), which also protects against diphtheria, tetanus, pertussis, *Haemophilus influenzae* type B, and polio [17]. In 2019, the provincial immunization committee proposed replacing the 18-month Infanrix-hexa dose with the bivalent Twinrix vaccine, which protects against hepatitis A and hepatitis B (HAHB), allowing for the introduction of a hepatitis A vaccination program for infants [18]. At the same time, the need for the second dose given at 4 months was questioned given the high efficacy of a 2-dose schedule and the very low prevalence of hepatitis B in Quebec and Canada. However, more data were needed, particularly because studies assessing 2-dose hepatitis B vaccination schedules in infants used a very short interval between doses, which differed from the schedule used in Quebec. The infant immunization schedule in Quebec does not include birth dose.

The objective of this study was to compare the immunogenicity of a homologous 2-dose schedule (Infanrix-hexa/Infanrix-hexa at 2 and 12 months), a heterologous 2-dose schedule (Infanrix-hexa/Twinrix at 2 and 12 months) and a homologous 3-dose schedule (Infanrix-hexa at 2, 4, and 18 months) in infants, with randomization of the 2 groups receiving the 2-dose schedule.

## METHODS

### Design and Study population

This open-label randomized clinical trial included 2 groups of infants randomized to receive 2 different 2-dose schedules and a nonrandomized control group that received a 3-dose schedule of hepatitis B vaccine. The study was conducted in the metropolitan area of Quebec City (province of Quebec, Canada), with recruitment from various pediatric vaccination centers. There was no patient or public involvement in the design, conduct, and reporting of the trial.

**Figure 1.**
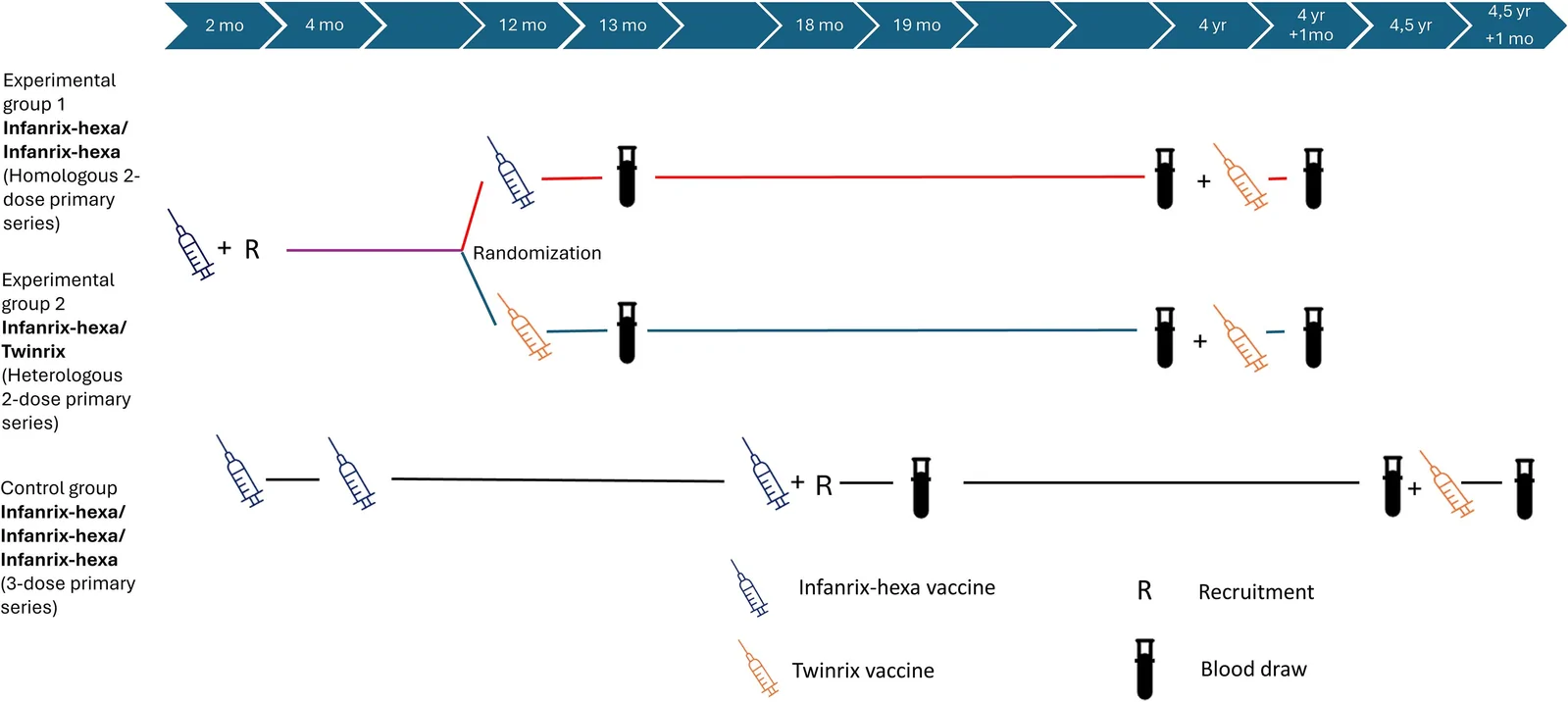
Study design. Experimental groups: first dose of the primary series at 2 months, second dose at 12 months. Control group: first dose of the primary series at 2 mo, second dose at 4 mo and third dose at 18 mo. These children had received all 3 doses before recruitment. For the 2- and 4-mo visit, an interval of 2 weeks before and 6 weeks after the scheduled date was accepted. For the 12-mo visit, a delay of 28 d was accepted. For the 18-mo visit, an interval of 2 weeks before and 12 weeks after the scheduled date was accepted. Challenge dose: Twinrix was administered 3 y ± 6 mo after the primary vaccination series. First blood draw: 33 ± 5 d after the primary vaccination series. Second blood draw: at the same visit as the challenge dose (sampling just before the administration of the challenge dose). Third blood draw: 33 ± 5 d after the challenge dose. Abbreviations: mo, months; y, years.

Children who received the first dose of the hexavalent vaccine Infanrix-hexa (DTaP-IPV-Hib-HB) at 2 months of age were randomized 1:1 using central computer to receive a second dose of 1 of 2 vaccines containing a hepatitis B component at 12 months of age, creating 2 experimental groups: Infanrix-hexa/Infanrix-hexa or homologous 2-dose (group 1) and Infanrix-hexa/Twinrix or heterologous 2-dose (group 2) (Figure 1). For the control group, 18-month-old children who had received 3 doses of the Infanrix-hexa vaccine according to the regular vaccination schedule in place in Quebec before 2019 were recruited (Infanrix-hexa/Infanrix-hexa/Infanrix-hexa or homologous 3-dose, group 3). An additional challenge dose (not part of the regular schedule) using the Twinrix vaccine was administered to children in all 3 groups 3 years ± 6 months after the primary series to mimic exposure to the virus and to assess immunological memory. This interval enabled school-entry vaccines to be offered without the need for an additional visit. Participants in the experimental groups were followed from July 2018 to December 2023, and those in the control group from June 2018 to August 2023. After recruitment, vaccination and blood draws were performed by a research nurse.

Exclusion criteria for the study included the following: (1) having received hepatitis B vaccine doses other than those scheduled, (2) being considered immunocompromised, (3) having an autoimmune disease, (4) having contraindications to HBV vaccination as defined by the Quebec Immunization Protocol [19], (5) having a bleeding disorder, (6) having a significant developmental delay, (7) having participated or planning to participate in other clinical studies involving vaccines or products not licensed in Canada, and (8) having had a serious clinical reaction to vaccines administered in the study.

### Vaccines, Dosage, and Schedules

Two hepatitis B vaccines licensed in Canada were used: the hexavalent vaccine Infanrix-hexa (DTaP-IPV-Hib-HB) and the combination vaccine Twinrix junior (HAHB), each containing 10 µg of HBsAg in a 0.5 mL single-dose syringe. Both vaccines are manufactured by GlaxoSmithKline (GSK). The other vaccines included in the Quebec schedule for the specific group were also administered during the study period (Table 1). The Quebec vaccination schedule was updated on 1 June 2019. The 3-dose group followed the infant schedule for those born before this date, while the 2-dose groups followed the schedule for those born after this date.

**Table 1.** Vaccines Administered to Participants

Age at Vaccination	Primary Series Schedule		
	Homologous two-dose	Heterologous two-dose	Three Doses
2 mo	Hexa (DTaP-IPV-Hib-**HB**) Pneumococcal Rotavirus	Hexa (DTaP-IPV-Hib-**HB**) Pneumococcal Rotavirus	Hexa (DTaP-IPV-Hib-**HB**) Pneumococcal Rotavirus
4 mo	Penta (DTaP-IPV-Hib) Pneumococcal Rotavirus	Penta (DTaP-IPV-Hib) Pneumococcal Rotavirus	Hexa (DTaP-IPV-Hib-**HB**) Pneumococcal Rotavirus
6 mo			Penta (DTaP-IPV-Hib)
12 mo	Hexa (DTaP-IPV-Hib-**HB**) Pneumococcal MMRV^a^	Bivalent (HA-**HB**) Penta (DTaP-IPV-Hib) Pneumococcal	Pneumococcal MMR Meningococcal C
13 mo		MMRV^a^	
18 mo	MMRV Meningococcal C	MMRV Meningococcal C	Hexa (DTaP-IPV-Hib-**HB**) MMRV
4 y^b^	Bivalent (HAHB)	Bivalent (HAHB)	
4, 5 y^c^			Bivalent (HAHB)

The hepatitis B component of vaccines is highlighted in bold text.

Abbreviations: DTaP-IPV-Hib-HB, diphtheria-tetanus toxoids-acellular pertussis-inactivated poliomyelitis-adsorbed conjugated *Haemophilus influenzae* type b-hepatitis B; MMRV, measles-mumps-rubella-varicella; HAHB, hepatitis A-hepatitis B.

^a^The main difference regarding other vaccines administered to children in the 2-dose groups relates to the MMRV vaccine. Children in the heterologous 2-dose group received this vaccine at 13 months of age instead of 12 months.

^b^Children have received dcaT-IPV, 30 days after HAHB, during third blood draw visit.

^c^Children have received dcaT-IPV and Varicella vaccine, 30 days after HAHB, during third blood draw visit.

### Immunogenicity assessment

Three blood draws were collected for each group (Figure 1). The first was collected 4 weeks after the last dose of the primary series: at 13 months for the homologous 2-dose and heterologous 2-dose experimental groups and at 19 months for the 3-dose control group. The second and third blood draws were collected immediately before and 4 weeks after administration of the challenge dose, respectively. Antibodies to hepatitis B surface antigen (anti-HBs) were measured by commercial enzyme immunoassay (MONOLISA Anti-HBs EIA#25220, MONOLISA Anti-HBs Calibrator Kit#25219, Bio-Rad Laboratory) according to the manufacturer's instructions [20]. The limit of quantification for testing was 5 mIU/mL. Seropositivity (anti-HBs ≥5 mIU/mL), seroprotection rate (anti-HBs ≥10 mIU/mL), and geometric mean titers (GMTs) were calculated for each blood draw. For GMTs calculation, a value of 1 unit was assigned to serological results below the limit of quantification as recommended previously [21].

A response to the challenge dose was defined as anti-HBs titers at least 10 mIU/mL and a 4-fold increase in titers 1 month after the challenge dose [22]. All tests were performed at the Quebec Public Health Laboratory (LSPQ).

### Assessment of reactogenicity

Local reactions (pain, redness, swelling, induration) and systemic reactions (irritability, drowsiness, sleep disturbance, decreased appetite, fever) were recorded by the nurse immediately after each vaccination at the research center, then by the parents for the following 5 days through diaries. Each reaction was coded as follows: grade 1 (does not interfere with daily activities), grade 2 (interferes with daily activities), and grade 3 (prevents daily activities). In the presence of redness, swelling, or induration, the largest diameter was coded as follows: grade 1 (1–*<*10 mm), grade 2 (10–*<*50 mm), or grade 3 (≥50 mm). Fever was defined as a body temperature above 37.4°C axillary, >37.9°C oral/tympanic, or >38.4°C rectal. Reported serious adverse events were recorded throughout the study period.

### Statistical analyses

The target sample size was 160 subjects per group. The goal was to have 145 evaluable subjects in each group at the end of the 3-year follow-up period, considering an estimated loss to follow-up of 2%–3% per year, 80% power, an alpha value of 5%, and a 4% difference in seroprotection between the 2-dose and 3-dose groups. The main analyses focused on participants who met all protocol requirements (per-protocol analysis), while the secondary analysis included all participants (intention-to-treat analysis). A descriptive analysis of participant characteristics was first performed, followed by assessments of seropositivity, seroprotection, GMTs, and response to the challenge dose. The primary outcomes were seroprotection after primary vaccination and response to the challenge dose (anti-HBs titers of at least 10 mIU/mL and a 4-fold increase in titers). A comparison between the 3 groups for each outcome was performed using Fisher's exact test (seropositivity and seroprotection) or analysis of variance (GMTs). A comparison of 2 specific groups was only presented when relevant. Multivariable analysis was done using log binomial regression in order to compare seroprotection rates or responses to the challenge dose, adjusting for the potentially confounding effect of sex, birth weight, and age at the first dose of the primary vaccination [13, 23, 24]. For the reactogenicity analysis, the frequency of local and systemic reactions reported by all participants was described and a comparison between study groups was performed using Fisher's exact test. All statistical analyses were performed using SAS software (SAS Institute, Cary, North Carolina, version 9.4). *P* values ≤ .05 were considered statistically significant.

### Ethics

This trial was registered (#NCT04294433) and approved by the research ethics committees of the *CHU de Québec-Université Laval* (#MP-20-2018-3992) and the *CIUSSS de la Capitale-Nationale* (#2017-2018-9022).

## RESULTS

### Participant Characteristics

**Figure 2.**
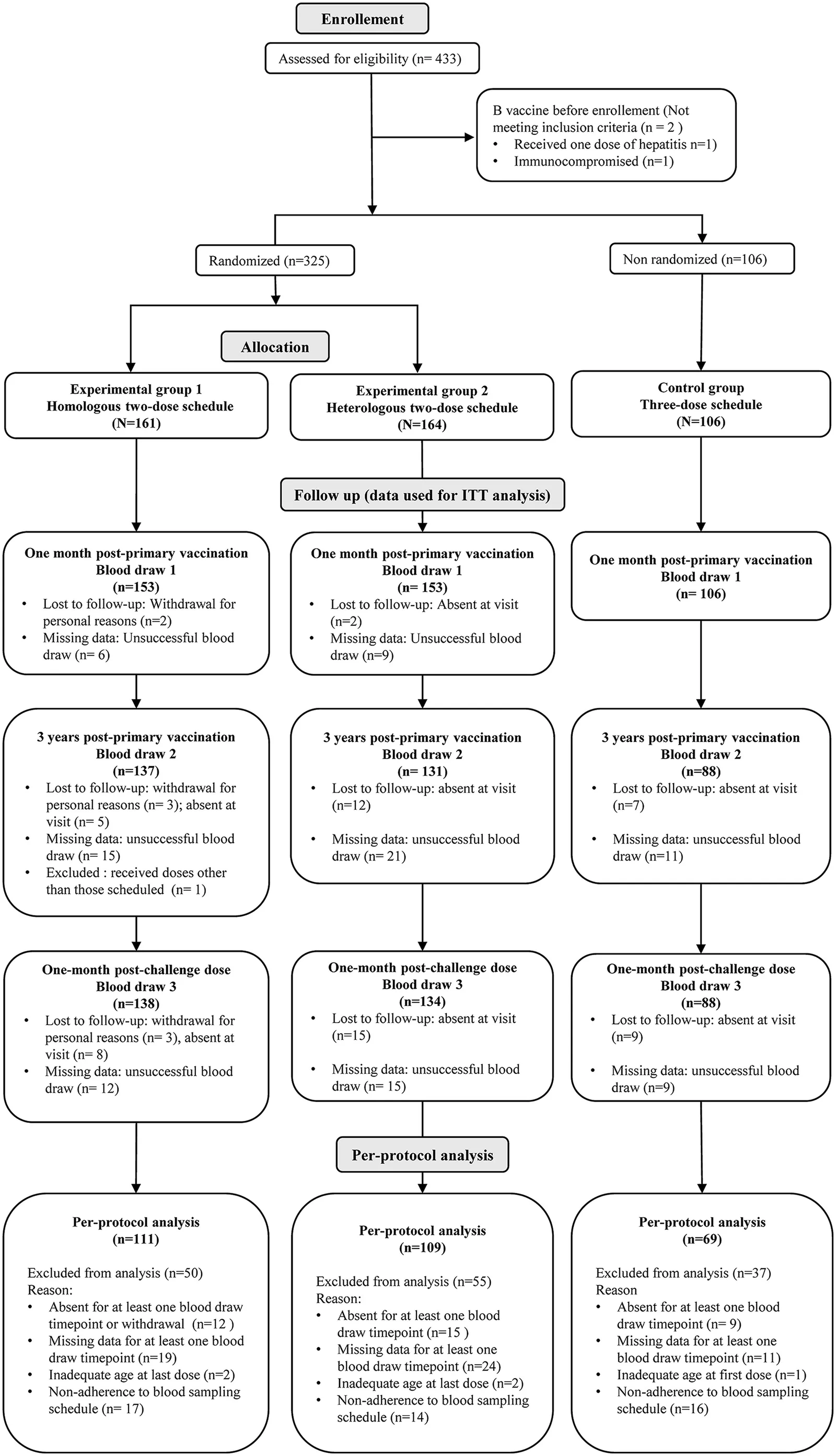
Flow chart of study participants. The homologous 2-dose schedule involves administering one dose of Infanrix-hexa at 2 mo of age, followed by a second dose at 12 mo. A challenge dose using the Twinrix vaccine was then given 3 y later. The heterologous 2-dose schedule involves administering one dose of Infanrix-hexa at 2 mo of age, followed by one dose of Twinrix at 12 mo. A challenge dose using the Twinrix vaccine was then given 3 y later. The 3-dose schedule involves administering 3 doses of Infanrix-hexa at 2, 4, and 18 mo of age. A challenge dose using the Twinrix vaccine was then given 3 y later. Eligibility criteria for the per protocol analysis: (1) having done all 3 blood draws, (2) interval between the last dose of primary vaccination and the first blood draw: 33 ± 5 d, (3) interval between the last dose of primary vaccination and the second blood draw: 3 y ±6 mo, (4) interval between the challenge dose and the third blood draw: 33 ± 5 d, (5) 2- and 4-mo vaccines given < 2 weeks before or < 4 weeks after the target age, (6) 12-mo vaccines given < 28 d after the target age, (7) 18-mo vaccines given < 2 weeks before and < 12 weeks after the target age. Abbreviations: ITT, Intention-to-treat analysis, including all participants.

Of the 433 recruited children, 431 were enrolled in the study. Of these, 325 were recruited at 2 months of age and randomized 1:1 in the homologous 2-dose group (n = 161) and the heterologous 2-dose group (n = 164). The remaining 106 children were recruited at 18 months to form the 3-dose control group. A total of 142 participants were excluded from the per-protocol analysis (homologous 2-dose [n = 50], heterologous 2-dose [n = 55] and 3-dose [n = 37]). Of these, 36 were excluded due to loss to follow-up for the primary endpoint (homologous 2-dose [n = 12], heterologous 2-dose [n = 15], and 3-dose [n = 9]). The remaining 106 participants were excluded due to missing data (n = 54) or nonadherence to the vaccination or blood sampling schedule (n = 52). Consequently, 289 participants were included in the per-protocol analysis: homologous 2-dose (n = 111), heterologous 2-dose (n = 109), and 3-dose (n = 69) (Figure 2).

The characteristics of the 2 experimental groups were comparable, except for a slightly higher proportion of males in the homologous 2-dose group (Table 2). Differences between the experimental groups and the control group were due to differences in the vaccination schedule and the recruitment date. Birth weight, age at first dose, and intervals between doses and blood draws were similar for all groups.

**Table 2.** Characteristics of Participants by Primary Hepatitis B Series Schedule, Per-Protocol Analysis

	Primary Hepatitis B Series Schedule		
	Homologous two-dose	Heterologous two-dose	Three Doses
	n = 111	n = 109	n = 69
Sex, n (%)			
Girls	46 (41.4)	54 (49.5)	34 (49.3)
Boys	65 (58.6)	55 (50.5)	35 (50.7)
Prematurity (<37 weeks), n (%)^a^	9 (8.1)	5 (4.6)	2 (2.9)
Birth weight, n (%)^b^			
Low birth weight (< 2500 g)	5 (5.2)	6 (6.1)	4 (9.8)
Normal birth weight (2500–4000 g)	80 (83.3)	82 (83.7)	34 (82.9)
High birth weight (> 4000 g)	11 (11.5)	10 (10.2)	3 (7.3)
Age at first dose, weeks			
Mean (SD)	9.3 (0.8)	9.2 (0.8)	9.1 (0.9)
Median (IQR)	9.1 (8.8–9.5)	9.1 (8.8–9.5)	9.0 (8.5–9.4)
Min–Max	6.0–12.1	7.4–13.5	6.4–12.4
Age at last dose of primary series, mo			
Mean (SD)	12.3 (0.2)	12.2 (0.2)	18.3 (0.4)
Median (IQR)	12.2 (12.1–12.4)	12.2 (12.1–12.4)	18.2 (18.0–18.5)
Min–Max	12.0–12.9	12.0–12.9	17.6–20.2
Age at first blood draw, mo			
Mean (SD)	13.3 (0.2)	13.3 (0.2)	19.4 (0.4)
Median (IQR)	13.2 (13.1–13.4)	13.2 (13.1–13.4)	19.2 (19.1–19.6)
Min–Max	12.9–14.0	12.9–13.9	18.5–21.1
Age at third blood draw, mo			
Mean (SD)	50.3 (1.0)	50.5 (1.0)	54.1 (3.8)
Median (IQR)	50.0 (49.6–50.8)	50.3 (49.8–50.8)	53.1 (50.7–57.6)
Min–Max	49.0–54.2	49.0–53.9	49.3–62.0
Interval between last dose and first blood draw, d			
Mean (SD)	31.6 (2.6)	31.4 (2.9)	32.1 (2.8)
Median (IQR)	32.0 (29.0–34.0)	31.0 (28.0–34.0)	32.0 (30.0–34.0)
Min–Max	28.0–38.0	28.0–38.0	28.0–38.0
Interval between last dose and second blood draw, mo			
Mean (SD)	37.1 (1.0)	37.2 (1.0)	34.7 (3.8)
Median (IQR)	36.8 (36.4–37.4)	37.0 (36.5–37.6)	33.7 (31.2–38.1)
Min–Max	35.3–40.9	35.4–40.6	30.2–41.9
Interval between challenge dose and third blood draw, d			
Mean (SD)	30.6 (2.8)	30.4 (2.8)	30.2 (3.0)
Median (IQR)	29.0 (28.0–33.0)	29.0 (28.0–33.0)	28.0 (28.0–33.0)
Min–Max	28.0–38.0	28.0–38.0	28.0–38.0

Abbreviations: g, grams; IQR, interquartile range; n, number of participants; SD, standard deviation.

^a^Missing data: n = 17 (homologous 2-dose group), n = 15 (heterologous 2-dose group), n = 28 (3-dose group).

^b^Missing data: n = 15 (homologous 2-dose group), n = 11 (heterologous 2-dose group), n = 28 (3-dose group).

### Immunogenicity (Per-Protocol Analysis)

One month after the primary vaccine series (second dose for the experimental groups and third dose for the control group), there was no statistically significant difference between the 3 groups for seroprotection (*P* > .8). The seroprotection rates (≥10 mIU/mL) were 90.9% (101/111) for the homologous 2-dose group, 91.7% (100/109) for the heterologous 2-dose group and 94.2% (65/69) for the 3-dose group. However, GMTs were significantly lower for both experimental groups compared to the control group: 360.2 mIU/mL (homologous 2-dose group), 570.6 mIU/mL (heterologous 2-dose group) and 1497.2 mIU/mL (3-dose group) (*P* = .002).

Three years after completion of the primary vaccination series, the seroprotection rates were 44.1%, 75.2%, and 76.8% for the homologous 2-dose, heterologous 2-dose, and 3-dose groups, respectively. There was no statistically significant difference between the heterologous 2-dose and the 3-dose groups (*P* > .8). GMTs were lowest in the homologous 2-dose group (6.5 mIU/mL), intermediate in the heterologous 2-dose group (38.0 mIU/mL) and highest in the 3-dose group (80.3 mIU/mL).

One month after the challenge dose, seroprotection rates were similar for the 3 groups (homologous 2-dose: 95.5%; heterologous 2-dose: 97.2%; 3-dose: 95.6%) (*P* > .6). The proportions of participants with a response to the challenge dose were also similar for all groups (homologous 2-dose: 95.5% (106/111), heterologous 2-dose: 97.2% (106/109), 3-dose: 92.7% (64/69)) (*P* = .36). GMTs were high for all 3 groups, with higher titers for the heterologous 2-dose group (9380.5 mIU/mL) compared to the 3-dose group (6900.6 mIU/mL), although the difference was not statistically significant (*P* = .41) (Table 3).

**Table 3.** Anti-HBs Antibody Responses by Vaccination Schedule, Per-Protocol Analyses

						
			Anti-HBs Antibodies			
Time of blood draw	Primary series schedule	n	% Seropositivity (≥5 mIU/mL) [95% CI]	% Seroprotection (≥10 mIU/mL) [95% CI]	%RCD [95% CI]	GMT, mIU/mL [95% CI]
Post-primary series blood draw^a^	Homologous 2-dose schedule	111	91.9 [85.1–96.2]	90.9 [84.0–95.6]		360.2 [221.2–586.3]*
	Heterologous 2-dose schedule	109	92.6 [86.0–96.8]	91.7 [84.9–96.1]	N/A	570.6 [347.7–936.4]*
	3-dose schedule	69	94.2 [85.8–98.4]	94.2 [85.8–98.4]		1497.2 [768.1–2918.5]*
Pre-challenge blood draw^b^	Homologous 2-dose schedule	111	52.2 [42.5–61.8]*	44.1 [34.7–53.8]*		6.5 [4.4–9.5]*
	Heterologous 2-dose schedule	109	82.5 [74.1–89.1]*	75.2 [66.0–83.0]*	N/A	38.0 [24.9–57.8]*
	3-dose schedule	69	84.0 [73.2–91.7]*	76.8 [65.1–86.1]*		80.3 [42.9–150.3]*
Post-challenge blood draw^c^	Homologous 2-dose schedule	111	96.4 [91.0–99.0]	95.5 [89.8–98.5]	95.5 [89.8–98.5]	1448.7 [927.2–2263.4]*
	Heterologous 2-dose schedule	109	97.2 [92.1–99.4]	97.2 [92.1–99.4]	97.2 [92.1–99.4]	9380.5 [6077.5–14 478.5]*
	3-dose schedule	69	95.6 [87.8–99.1]	95.6 [87.8–99.1]	92.7 [83.9–97.6]	6900.6 [3645.7–13 061.5]*

Abbreviations: CI, confidence interval; GMT, geometric mean titer; n, number of participants; N/A, not applicable; RCD, response to challenge dose (defined as at least 10 mIU/mL and a 4-fold increase in anti-HB titer one month after the challenge dose using Twinrix).

^a^Blood draw 33 ± 5 days after the last dose of the primary vaccination series.

^b^Blood draw 3 years ± 6 months after the last dose of the primary vaccination series.

^c^Blood draw 33 ± 5 days after the challenge dose.

^*^ *P* value (comparison of the 3 groups) < .05.

The results were similar in the multivariable analysis, which adjusted for certain potential confounding factors. There was no statistically significant difference between the 3 vaccination schedules for the seroprotection rate after primary vaccination (*P*_crude_ = .82; *P*_adjusted_ = .84) and for the response to the challenge dose (*P*_crude_ = .37; *P*_adjusted_ = .44) (data not shown). The conclusions were also similar in the intention-to-treat analyses (Supplementary Tables 1 and 2).

Of the 289 participants included in the per-protocol analysis, 23 (8%) did not reach the seroprotection threshold (10 mIU/mL) one month after the primary series. Sixteen had no detectable antibodies (0 mIU/mL), representing 5% of the total cohort. Three years after the primary vaccination, 15/23 children (65%) who had previously not reached the seroprotection threshold responded to the challenge dose (7/10 for the homologous 2-dose group, 6/9 for the heterologous 2-dose group and 2/4 for the 3-dose group; *P* > .8). Among participants who did not have detectable antibodies after primary vaccination, 9/16 responded to the challenge dose. For participants who had detectable antibodies but did not reach the seroprotection threshold after primary vaccination (<10 mIU/mL), all except one (6/7) responded to the challenge dose (Supplementary Table 3).

### Reactogenicity

A total of 317 parents completed the vaccination diary after primary vaccination in the experimental groups (homologous 2-dose, 157; heterologous 2-dose, 160) and 390 diaries were collected after the challenge dose (homologous 2-dose, 149; heterologous 2-dose, 146; 3-dose, 95). The incidence of local reactions reported after the primary series was significantly higher in the homologous 2-dose experimental group than in the heterologous 2-dose experimental group (pain: 47.7% vs 30.6%; redness: 43.9% vs 24.3%, swelling: 20.3% vs 5.0%; induration, 26.7% vs 7.5%) (*P* < .002; Table 4). Grade 3 reactions were rare in both groups. There were no statistically significant differences between the groups for systemic reactions after the primary series or for local and systemic reactions after the challenge dose. There were no serious adverse events recorded.

**Table 4.** Solicited Local and Systemic Reactions Following the Primary Series and Challenge Dose, by Vaccination Schedule

	Post-Primary Series			Post-Challenge Dose			
Reactions	Homologous two-dose (n = 157) % [95% CI]	Heterologous two-dose (n = 160) % [95% CI]	*P* value	Homologous two-dose (n = 149) % [95% CI]	Heterologous two-dose (n = 146) % [95% CI]	Three doses (n = 95) % [95% CI]	*P* value
Local reactions							
Pain	47.7 [39.7–55.8]	30.6 [23.6–38.4]	.**0019**	34.9 [27.2–43.1]	40.4 [32.3–48.8]	40.0 [30.0–50.5]	.57
Grade 3^a^	0.0 [0.0–2.3]	0.6 [0.0–3.4]		0.0 [0.0–2.4]	0.0 [0.0–2.5]	0.0 [0.0–3.8]	
Redness	43.9 [36.0–52.1]	24.3 [17.9–31.8]	.**0002**	18.8 [12.8–26.0]	18.5 [12.5–25.7]	21.0 [13.3–30.6]	.87
Grade 3	2.5 [0.7–6.4]	0.0 [0.0–2.2]		0.0 [0.0–2.4]	0.0 [0.0–2.5]	0.0 [0.0–3.8]	
Swelling	20.3 [14.3–27.5]	5.0 [2.1–9.6]	**<**.**0001**	4.7 [1.9–9.4]	8.9 [4.8–14.7]	6.3 [2.3–13.2]	.34
Grade 3	2.5 [0.7–6.4]	0.0 [0.0–2.2]		0.0 [0.0–2.4]	0.0 [0.0–2.5]	0.0 [0.0–3.8]	
Induration	26.7 [20.0–34.4]	7.5 [3.9–12.7]	**<**.**0001**	8.0 [4.2–13.6]	9.6 [5.3–15.5]	13.7 [7.4–22.2]	.36
Grade 3	0.6 [0.0–3.5]	0.0 [0.0–2.2]		0.0 [0.0–2.4]	0.0 [0.0–2.5]	1.0 [0.0–5.7]	
Systemic reactions							
Irritability	78.3 [71.0–84.5]	76.8 [69.5–83.2]	.78	30.2 [22.9–38.2]	32.8 [25.3–41.1]	32.6 [23.3–43.0]	.89
Grade 3	5.7 [2.6–10.6]	3.1 [1.0–7.1]		1.3 [0.1–4.7]	0.0 [0.0–2.5]	1.0 [0.0–5.7]	
Drowsiness	54.1 [46.0–62.2]	50.6 [42.6–58.6]	.57	17.4 [11.7–24.5]	17.8 [11.9–24.9]	15.8 [9.1–24.7]	.93
Grade 3	0.6 [0.0–3.5]	1.8 [0.4–5.3]		0.6 [0.0–3.6]	0.0 [0.0–2.5]	0.0 [0.0–3.8]	
Sleep disturbance	63.7 [55.6–71.2]	56.8 [48.8–64.6]	.25	10.7 [6.2–16.8]	20.5 [14.3–28.0]	14.7 [8.3–23.5]	.07
Grade 3	2.5 [0.7–6.4]	3.1 [1.0–7.1]		2.0 [0.4–5.7]	0.0 [0.0–2.5]	0.0 [0.0–3.8]	
Decreased appetite	55.4 [47.2–63.3]	50.0 [42.0–58.0]	.37	14.7 [9.4–21.5]	13.7 [8.5–20.4]	22.1 [14.2–31.8]	.20
Grade 3	1.2 [0.1–4.5]	1.8 [0.4–5.3]		0.0 [0.0–2.4]	0.0 [0.0–2.5]	0.0 [0.0–3.8]	
Fever^b^	22.9 [16.6–30.3]	25.0 [18.5–32.4]	.69	10.1 [5.7–16.1]	10.9 [6.4–17.2]	4.2 [1.1–10.4]	.16
Grade 3^c^	0.6 [0.0–3.5]	0.6 [0.0–3.4]		0.0 [0.0–2.4]	0.7 [0.0–3.7]	1.0 [0.0–5.7]	
Other reactions^d^	23.5 [17.1–30.9]	26.8 [20.1–34.4]	.52	19.4 [13.4–26.7]	15.7 [10.2–22.7]	18.9 [11.6–28.3]	.69

Statistically significant *P*-values are highlight in bold text.

Abbreviations: n, total number of subjects who completed the vaccination diary.

^a^Grade 3 (redness, swelling, induration), largest diameter ≥ 50 mm; Grade 3 (pain, irritability, drowsiness, sleep disturbance, decreased appetite), prevents daily activities.

^b^Fever: Axillary temperature ≥ 37.5°C or oral/tympanic temperature ≥ 38°C or rectal temperature ≥ 38.5°C.

^c^Grade 3 fever: Axillary temperature > 39°C or oral/tympanic temperature > 39.5°C or rectal temperature > 40°C.

^d^Other reactions: The most common reactions were vomiting, diarrhea, runny nose, headaches, and skin rashes that resolved spontaneously.

## DISCUSSION

This study showed that an off-label schedule consisting of 2 doses of hepatitis B vaccine at 2 and 12 months of age appeared as immunogenic as a 3-dose schedule at 2, 4, and 18 months of age, based on seroprotection rates after the primary series and response to a challenge dose administered 3 years later. Both the 2-dose and 3-dose schedules provided seroprotection to over 90% of participants and a strong anamnestic response after a challenge dose. In addition, the heterologous 2-dose schedule was associated with stronger immunogenicity and lower reactogenicity than the homologous 2-dose schedule.

The results obtained 1 month after the 2-dose primary series are consistent with other studies that used a reduced-dose hepatitis B vaccination schedule during the first year of life. Akram et al [12]. published a study in 2005 in which they administered 2 doses of a recombinant hepatitis B vaccine (Heberbiovac HB, Heber Biotec, Havana, 10 µg) to 117 Pakistani children. They found a very high seroprotection rate of 98%, but this result is difficult to interpret because the exact age at vaccination was not specified. In another study conducted in Italy, the seroprotection rate was 97.5% among 163 children vaccinated against hepatitis B at 3 and 5 months of age and lower (58.7%) among 46 children who received the same vaccine at 1 and 3 months of age [11]. Similar results were found in other studies, and the immunogenicity of a 2-dose schedule starting at birth or at one month of age was considered insufficient [25]. According to our study results, vaccination at 2 and 12 months seems to occur late enough to provide adequate seroprotection, similar to a 3-dose schedule given at 2, 4, and 18 months of age.

When we compare our seroprotection data obtained after primary vaccination with the data from the aforementioned studies [11, 12, 25], we generally find lower values in our study. Our immunoassay (MONOLISA Anti-HBs EIA) was less sensitive (94.2%) than other assays but highly specific (97.7%) [26]. This could partly explain the lower seroprotection rates (<95%) obtained for our 3 groups.

The use of a challenge dose 3 years after the primary series mimicked exposure to the HBV. In the present study, the anamnestic response was strong and similar between groups. In particular, the heterologous 2-dose schedule administered at 2 and 12 months stood out due to significantly higher GMTs when compared to the homologous 2-dose schedule after the challenge dose (9381 and 1449 mIU/mL, respectively, *P* < .0001). Our findings align with those of previous studies that observed higher GMTs with combined HAHB vaccines than with the corresponding monovalent formulations [27]. The favorable immunogenic profile of the heterologous 2-dose schedule, both after the primary series and the challenge dose, could be explained by the fact that the Twinrix vaccine is highly immunogenic, as demonstrated by its performance in various previous studies [4, 14, 15]. This property is thought to be related to the synergistic action between the HAHB vaccines [28]. This may be biologically explained by a positive bystander effect, in which the HAV antigen acts as an immunological adjuvant for HBsAg. By delivering both components to the same injection site, the HAV-specific cell-mediated response is thought to enhance the recruitment and activation of HBV-specific T cells [29, 30].

Aligned with the literature, the 3 schedules studied showed a good safety profile [5, 31–35]. Once again, the heterologous 2-dose primary series stood out from the homologous 2-dose schedule with a better safety profile, namely a lower frequency of local reactions after Twinrix compared with Infanrix-hexa at 12 months of age. Previous studies had shown that Infanrix-hexa given in the second year of life may cause an increased number of significant local reactions [36, 37]. We cannot rule out the possibility that the lower local reactogenicity of Twinrix at 12 months compared to Infanrix-hexa is due to the co-administered vaccines. However, it is unlikely, as the Infanrix-hexa vaccine was administered alongside the MMRV vaccine, a combination that does not increase the risk of local reactions [37].

Most children who did not reach the seroprotection threshold after the primary series responded well to the challenge dose. This good response observed is reassuring regarding potential protection of these children in case of exposure to the virus. For diseases with an incubation period spanning several weeks to months, such as hepatitis B, a marked increase of anti-HBs after the challenge dose (anamnestic response) indicates memory cell stimulation and rapid antibody production that may prevent infection [38, 39]. In addition, studies have shown protection in individuals with low antibody titers, consistent with good immune memory [40, 41]. Our data are also consistent with earlier studies reporting a favorable response to an additional dose in individuals who had low antibody levels (<10 mIU/mL) after a primary series [42].

The data from this study have public health implications. Funding pediatric vaccination programs is a major challenge for many countries [43]. If a 2-dose schedule (homologous or heterologous) in infants provides protection equivalent to a 3-dose schedule, significant financial savings could be achieved, facilitating the potential introduction of new vaccines into public programs. In addition, the use of a heterologous schedule that includes Twinrix as the second dose has the advantage of providing protection against hepatitis A and may reduce reactogenicity. Several jurisdictions have successfully integrated a single-dose hepatitis A vaccination program [19, 44, 45], and such a schedule is now recommended by the WHO [46]. A cost-effectiveness analysis would be necessary to better evaluate a 2-dose regimen for infants, as well as a study to gather the opinions of parents and healthcare workers on such a schedule.

A key limitation of this study is the absence of randomization for the 3-dose group, which may lead to significant selection or confounding biases. This includes the fact that the children in the 3-dose group were recruited at 18 months of age instead of 2 months, meaning they were from a slightly older age cohort than those in the 2-dose groups. However, all groups were comparable for most characteristics, and the conclusions remained unchanged after multivariable adjustment for certain potential confounders. Second, a major limitation is that pre-vaccination serology was not performed to avoid inconveniencing participants and minimizing study costs. Reassuringly, the incidence of hepatitis B is extremely low in Quebec, particularly among individuals under the age of 10, at less than 1/100 000 person-years in 2022 [47]. Therefore, it is highly unlikely that the immune response was attributable to a previous natural HBV infection. Third, participants with a 2-dose schedule received the second dose at 12 months, which may partly explain the lower GMTs after the primary series compared to the 3-dose schedule, where the third dose was administered at 18 months. It could be expected that a 2-dose schedule offered at 2 and 18 months would result in even higher antibody titers. More data are needed regarding 2-dose schedules administered at different ages (eg, 2 and 18 months of age). Fourth, the sample size was relatively small and did not allow for the identification of very small differences in seroprotection between groups. However, the fact that the highest GMTs after the challenge dose were observed for the heterologous 2-dose schedule is reassuring regarding the potential protection conferred by this 2-dose primary series. Studies with a larger sample size would be helpful to exclude a small difference in seroprotection between groups. Fifth, although there were not enough children with low birth weight to conduct a specific analysis (n = 15), all of them responded to the challenge dose. However, the small sample size limits the generalizability of these findings. Sixth, there were notable differences between the 3 groups regarding co-administered vaccines. Reassuringly, several studies have shown that administering childhood vaccines concurrently does not interfere with the immune response to hepatitis B vaccines [48]. Finally, none of the schedules considered in our study included the birth dose recommended by the WHO [2]. A birth dose was not considered necessary in Quebec or Canada because the incidence of hepatitis B is very low [47]. Due to differences in schedules, our results cannot be applied to settings where a birth dose has been implemented.

In conclusion, a 2-dose hepatitis B vaccination schedule administered at 2 and 12 months appears to elicit immunogenicity equivalent to a 3-dose schedule, particularly when a heterologous Infanrix-hexa/Twinrix regimen is used. Such a schedule also has the added benefit of protecting against hepatitis A while reducing reactogenicity. Studies with longer follow-up periods are needed to confirm the sustainability of the 2-dose regimen's responses.

## Supplementary Material

jiag122_Supplementary_Data

## Supplementary Data

Supplementary materials are available at *The Journal of Infectious Diseases* online (http://jid.oxfordjournals.org/). Supplementary materials consist of data provided by the author that are published to benefit the reader. The posted materials are not copyedited. The contents of all supplementary data are the sole responsibility of the authors. Questions or messages regarding errors should be addressed to the author.
