# Supplementary material for: Immunogenicity of Two Versus Three Doses of Hepatitis B Vaccine When Administered to Children Aged 2–18 Months: A Randomized Clinical Trial
Source: J Infect Dis. 2026 Feb 25;234(2):e243–53. doi: 10.1093/infdis/jiag122 (PMC13537124; doi:10.1093/infdis/jiag122)
Supplement: Supplementary file 1 [file jiag122_supplementary_data.docx]

**SUPPLEMENTARY MATERIAL**

**Immunogenicity of two versus three doses of hepatitis B vaccine when administered to children aged 2-18 months: a randomized clinical trial.**

**Supplementary Table 1**. Characteristics of participants by primary hepatitis B series schedule, intention-to-treat analysis

|  | Primary series schedule | | |
| --- | --- | --- | --- |
|  | Homologous two-dose | Heterologous two-dose | Three doses |
|  | n =161 | n =164 | n =106 |
| **Sex, n (%)** |  |  |  |
| Girls | 66 (41) | 80 (49) | 54 (51) |
| Boys | 95 (59) | 84 (51) | 52 (49) |
| **Prematurity (<37 weeks), n (%)^a^** | 11 (6.8) | 8 (4.9) | 3 (2.8) |
| **Birth weight, n (%)^b^** |  |  |  |
| Low birth weight (< 2500g) | 5 (3.6) | 8 (5.4) | 4 (6.1) |
| Normal birth weight (2500 - 4000g) | 119 (85.6) | 126 (85.1) | 55 (83.3) |
| High birth weight (> 4000g) | 15 (10.8) | 14 (9.5) | 7 (10.6) |
| **Age at first dose, weeks** |  |  |  |
| Mean (SD) | 9.3 (0.7) | 9.2 (0.7) | 9.3 (2.1) |
| Median (IQR) | 9.1 (8.8 - 9.5) | 9.1 (8.8 - 9.5) | 9.0 (8.7 - 9.5) |
| Min-Max | 6.0 - 12.1 | 7.4 - 13.5 | 6.4 - 29.4 |
| **Age at last dose of primary series, months** |  |  |  |
| Mean (SD) | 12.3 (0.2) | 12.2 (0.2) | 18.3 (0.4) |
| Median (IQR) | 12.2 (12.1 - 12.5) | 12.2 (12.1 - 12.4) | 18.3 (18.0 - 18.5) |
| Min-Max | 12.0 - 13.4 | 11.0 - 13.0 | 17.4 - 20.2 |
| **Age at first blood draw, months** |  |  |  |
| Mean (SD) | 13.4 (0.4) | 13.3 (0.3) | 19.4 (0.4) |
| Median (IQR) | 13.3 (13.1 - 13.5) | 13.3 (13.1 - 13.5) | 19.3 (19.1 - 19.7) |
| Min-Max | 12.9 - 16.2 | 12.1 - 14.2 | 18.3 - 21.1 |
| **Age at third blood draw, months** |  |  |  |
| Mean (SD) | 50.3 (0.9) | 50.5 (1.0) | 53.9 (3.9) |
| Median (IQR) | 50.1 (49.6 - 50.8) | 50.3 (49.7 - 50.9) | 52.9 (50.6 - 57.3) |
| Min-Max | 49.0 - 54.3 | 49.0 - 53.9 | 49.3 - 62.0 |
| **Interval between last dose and first blood draw, days** |  |  |  |
| Mean (SD) | 33.3 (8.9) | 32.8 (5.4) | 34.1 (7.1) |
| Median (IQR) | 32.0 (29.0 - 35.0) | 32.0 (29.0 - 35.0) | 33.0 (29.0 - 35.0) |
| Min-Max | 22.0 - 121.0 | 28.0 - 57.0 | 27.0 - 71.0 |
| **Interval between last dose and second blood draw, months** |  |  |  |
| Mean (SD) | 37.1 (1.0) | 37.3 (1.1) | 34.5 (3.7) |
| Median (IQR) | 36.8 (36.4 - 37.4) | 37.0 (36.5 - 37.7) | 33.8 (31.2 - 37.9) |
| Min-Max | 35.4 - 40.9 | 35.4 - 40.6 | 29.5 - 41.9 |
| **Interval between challenge dose and third blood draw, days** |  |  |  |
| Mean (SD) | 30.7 (3.2) | 31.0 (3.9) | 31.3 (5.5) |
| Median (IQR) | 29.0 (28.0 - 33.0) | 29.0 (28.0 - 33.0) | 29.0 (28.0 - 33.5) |
| Min-Max | 26.0 - 42.0 | 28.0 - 50.0 | 27.0 - 58.0 |

Abbreviations: g, gram; IQR, interquartile range; n, number of participants; SD, standard deviation.

^a^ Missing data, n=24 (homologous two-dose), n=23 (heterologous two-dose), n=41(three-dose).

^b^ Missing data, n=22 (homologous two-dose), n=16 (heterologous two-dose), n=40 (three-dose).

**Supplementary Table 2.** Anti-HBs antibody responses by vaccination schedule, intention to treat analyses

|  |  |  | Anti-HBs antibodies | | | |
| --- | --- | --- | --- | --- | --- | --- |
| Time of blood draw | Primary series schedule |  | % Seropositivity  (≥5 mIU/mL)  [95% CI] | % Seroprotection  (≥10 mIU/mL)  [95% CI] | %RCD  [95% CI] | GMT, mIU/mL  [95% CI] |
|  |  | n |  |  |  |  |
| Post-primary  series blood draw^a^ | Homologous two-dose schedule | 153 | 90.2 [84.3 – 94.4] | 88.9 [82.8 – 93.4] |  | 305.5 [198.5 - 470.4] * |
|  | Heterologous two-dose schedule | 153 | 93.4 [88.3 - 96.8] | 92.8 [87.5 - 96.3] | N/A | 573.4 [383.7 - 856.8] * |
|  | Three-dose schedule | 106 | 96.2 [90.6 - 98.9] | 96.2 [90.6 - 98.9] |  | 1894.2 [1158.0 - 3098.4] * |
| Pre-challenge  blood draw^b^ | Homologous two-dose schedule | 137 | 52.5 [43.8 - 61.1] * | 43.1 [34.6 - 51.8] * |  | 6.6 [4.7 - 9.4] * |
|  | Heterologous two-dose schedule | 131 | 81.6 [73.9 - 87.9] * | 75.5 [67.3 - 82.6] * | N/A | 39.1 [26.3 - 58.1] * |
|  | Three-dose schedule | 88 | 86.3 [77.4 - 92.7] * | 80.7 [70.8 - 88.3] * |  | 93.9 [54.8 - 161.0] * |
| Post-challenge  blood draw^c^ | Homologous two-dose schedule | 138 | 96.4 [91.7 - 98.8] | 95.6 [90.7 - 98.4] | 95.5 [90.6 - 98.3] | 1531.2 [1024.3 - 2289.0] * |
|  | Heterologous two-dose schedule | 134 | 97.7 [93.6 - 99.5] | 97.7 [93.6 - 99.5] | 96.9 [92.2 - 99.1] | 9634.4 [6632.4 - 13995.1] * |
|  | Three-dose schedule | 88 | 96.6 [90.3 - 99.3] | 96.6 [90.3 - 99.3] | 94.2 [86.9 - 98.1] | 7589.1 [4471.5 - 12880.4] * |

Abbreviations: CI, confidence interval; GMT, geometric mean titer; n, number of participants; N/A, not applicable; RCD, response to challenge dose (defined as at least 10 mIU/mL and a fourfold increase in anti-HB titer one month after the challenge dose using Twinrix).

^a^ Blood draw 33 ± 5 days after the last dose of the primary vaccination series.

^b^ Blood draw 3 years ± 6 months after the last dose of the primary vaccination series.

^c^ Blood draw 33 ± 5 days after the challenge dose.

* p-value (comparison of the three groups) < 0.05.

**Supplementary Table 3.** Response to challenge dose among participants who did not reach seroprotection threshold (10mIU/mL) after primary series (n=23), per protocol analysis, for **(A)** all vaccination schedules, **(B)** homologous two-dose schedule, **(C)** heterologous two-dose schedule, and **(D)** three-dose schedule.

A) All vaccination schedules (n=23/289)

| Titers after the primary series (mIU/mL) | n | Response to challenge dose | | | | |
| --- | --- | --- | --- | --- | --- | --- |
|  |  | 0 | 0.1 - 9.9 | 10 - 99.9 | 100 - 999.9 | ≥ 1000 |
| 0 | 16 | 5 | 2 | 1 | 7 | 1 |
| 0.1 - 4.9 | 6 | 1 |  | 1 | 2 | 2 |
| 5 - 9.9 | 1 |  |  |  |  | 1 |

B) Homologous two-dose schedule (n=10/111)

| Titers after the primary series (mIU/mL) | n | Response to challenge dose | | | | |
| --- | --- | --- | --- | --- | --- | --- |
|  |  | 0 | 0.1 - 9.9 | 10 - 99.9 | 100 - 999.9 | ≥ 1000 |
| 0 | 7 | 1 | 2 | 1 | 3 | 2 |
| 0.1 - 4.9 | 3 |  |  | 1 |  |  |
| 5 - 9.9 | 0 |  |  |  |  |  |

C) Heterologous two-dose schedule (n=9/109)

| Titers after the primary series (mIU/mL) | n | Response to challenge dose | | | | |
| --- | --- | --- | --- | --- | --- | --- |
|  |  | 0 | 0.1 - 9.9 | 10 - 99.9 | 100 - 999.9 | ≥ 1000 |
| 0 | 7 | 3 |  |  | 3 | 1 |
| 0.1 - 4.9 | 1 |  |  |  | 1 |  |
| 5 - 9.9 | 1 |  |  |  |  | 1 |

D) Three-dose schedule (n=4/69)

| Titers after the primary series (mIU/mL) | n | Response to challenge dose | | | | |
| --- | --- | --- | --- | --- | --- | --- |
|  |  | 0 | 0.1 - 9.9 | 10 - 99.9 | 100 - 999.9 | ≥ 1000 |
| 0 | 2 | 1 |  |  | 1 |  |
| 0.1 - 4.9 | 2 | 1 |  |  | 1 |  |
| 5 - 9.9 | 0 |  |  |  |  |  |
